# Stability analysis and numerical evaluations of a COVID-19 model with vaccination

**DOI:** 10.1186/s12874-024-02209-2

**Published:** 2024-04-27

**Authors:** Mohammad Izadi, Tayebeh Waezizadeh

**Affiliations:** 1 Department of Applied Mathematics, Faculty of Mathematics and Computer, Shahid Bahonar University of Kerman, Kerman, Iran; 2 Department of Pure Mathematics, Faculty of Mathematics and Computer, Shahid Bahonar University of Kerman, Kerman, Iran; 3 Mahani Mathematical Research Center, Shahid Bahonar University of Kerman, Kerman, 76169-14111 Iran

**Keywords:** Collocation points, Stability analysis, Convergent analysis, COVID-19 model, Chebyshev functions, 34A34, 65L60, 41A10, 65L20, 34D20, 34C11

## Abstract

A novel (nonlinear) mathematical model for the transmission of Coronavirus 19 (COVID-19) with eight compartments and considering the impact of vaccination is examined in this manuscript. The qualitative behavior of the system such as the boundedness of solutions, the basic reproduction number, and the stability of the equilibrium points is investigated in detail. Some domestic real data collected from the Kerman University of Medical Science (KUMC) is used to estimate the parameters of the proposed model. We predict the dynamical behavior of the system through numerical simulations based on a combined spectral matrix collocation methodology. In this respect, we first linearize the nonlinear system of equations by the method of quasilinearization (QLM). Hence, the shifted version of Chebyshev polynomials of the second kind (SCPSK) is utilized along with the domain-splitting strategy to acquire the solutions of the system over a long time interval. The uniform convergence and upper bound estimation of the SCPSK bases are proved in a rigorous manner. Moreover, the technique of residual error functions is used to testify the accuracy of the QLM-SCPSK method. The presented numerical results justify the robustness and good accuracy of the QLM-SCPSK technique. The achieved numerical orders of convergence indicate that the QLM-SCSK algorithm has exponential rate of convergence. Using the linearization technique in one hand and the domain-splitting strategy on the other hand, enable us to predict the behaviour of similar disease problems with high accuracy and maximum efficiency on an arbitrary domain of interest.

## Introduction

Infectious diseases have always been a limitation of human life. Many epidemics throughout history have killed millions of people. The last of them was the COVID-19 virus, the first case of which was observed in China in December 2019. The disease quickly spread throughout the world within a few months. In the mid of Mach 2020, WHO announced that the infection was an outbreak. A prominent feature of the current pandemic is the high person-to-person transmissibility of the virus, with a basic reproduction number $(R_0)$ estimated at 2.2-2.5 in Wuhan, China. This caused an increase in hospitalizations and as a result deaths due to the disease [1, 2]. Researchers in the field of epidemiology and other branches of biology tried to find treatments or vaccines for this disease. The first vaccines were clinically tested at the beginning of 2021. In the following months, a number of highly effective vaccines entered the market. Mathematical models can describe the process of transmission of infection and its control using vaccination.

Researchers try to understand the dynamics of a disease in the first stage. Hence, they develop control and curing procedures for the diseases. The first modern mathematical models were introduced by Kermak and Mckendrick in 1927 [3]. Since 2019, many mathematical models have been presented. Different aspects of the novel COVID-19 have been investigated through mathematical models [4–6]. Before vaccine production, the effect of quarantine and control factors were investigated [7, 8].

Butt et al. developed and analyzed mathematically the extended SEQIHR model. The authors determined the possible control strategies and comprehended the long-term dynamics of disease [9]. A SEQIER model introduced to examine the transmission dynamics of COVID-19. The focus is on the effectiveness of hospitalization and quarantine strategies [10, 11]. A deterministic SEIHR fractional model is developed in [12]. The authors performed the mathematical analysis and the design of an optimal control strategy for the proposed Caputo-Fabrizio fractional model. In [13], the authors analyzed a fractional order initial value problem with the Atangana-Baleanu derivative operator to observe transmission dynamics of the infection in a human population. An SVEIR mathematical model was introduced to predict transmission dynamics of COVID-19 infection [14]. In [15], the effect of virus rate in the environment in a deterministic model is investigated. Some of the mathematical models of COVID-19 are presented by fractional-order models [16].

Vaccination is very effective to control infectious diseases. Therefore, after producing vaccines for coronavirus, researchers developed dynamical models regarding the effectiveness of vaccines. The authors in [17] developed vaccination strategies for vaccination and investigated the dynamic of their epidemic model. A dynamical model considering the treatment and vaccination saturating function is introduced in [18]. The study of the vaccination model under vaccine immunization has been considered in [19]. The concerned investigations are devoted to stability theory, numerical simulation, and global-local dynamics [20]. One of the items that is investigated in the dynamic analysis of models is bifurcation. Researchers have investigated the existence of bifurcation in different models [21–23].

In this paper, a (novel) mathematical model for COVID-19 with an omicron version is presented. In this model, vaccination and its effect on people is considered. In this study, the importance of vaccination to control the epidemic has been investigated. In this model, vaccinated people and those who have not received the vaccine are divided into two groups. Therefore, the rate of infection and hospitalization rate in each group is investigated separately. In this deterministic model, the population is divided into two main parts, people who have been vaccinated and people who have not received any vaccine. It is also assumed that recovered individuals are prepared to reinfection after some time. As follows, some basic dynamic properties of the proposed model are investigated. Using the statistics of the Ministry of Health of Iran in Kerman province, the parameters are estimated over a period of time.

Furthermore, we develop a combined spectral collocation approach based on the shifted version of Chebyshev polynomials of the second kind (SCPSK) to predict the behaviour of COVID-19 disease. To get efficacy, we first transform the nonlinear system of ODEs into a set of linearized system of eight equations to be treated iteratively. Hence, the employed SCPSK basis functions together with the domain decomposition strategy are utilized to find the solutions of the linearized systems. We also prove the convergence of the SCPSK bases and an upper bound estimation for them is performed. It should be noted that spectral-based collocation techniques have been used often to tackle various integral and differential equations due to their simplicity of implementations and high-order accuracy. These spectral methods have been benefited by utilizing numerous basis functions like the Vieta-Lucas [24], Chebyschev [25–27], Bessel [28, 29], Chelyshkov [30–32], and the Schröder [33] polynomials.

Let us illustrate the main achievements of the present work in a concise form as follow:

- A novel mathematical model consists of eight equations for the studying of COVID-19 transmission is proposed in which the effect of vaccination is considered.
- The parameters of the model are estimated from the real data provided by the KUMC.
- The dynamic analysis of the proposed COVID-19 model is performed from theoretical points of stability, boundedness, and the existence of bifurcations.
- A combined efficient method called the QLM-SCPSK algorithm based on the technique of quasilinearization along with the Chebyshev spectral collocation approach is designed to solve the given model numerically and confirm the theoretical findings as well.
- The strategy of domain-splitting is further employed to keep the accuracy of the proposed QLM-SCPSK algorithm at a desired level. The exponential convergence of the employed SCPSK bases in the infinity norm is established in a rigourous analysis.

The structure of the current work is provided as follows. A mathematical model considering vaccination is introduced and some basic dynamical properties such as the boundedness of solutions, the derivation of the basic reproduction number, the stability of the equilibrium points, and the existence of bifurcation are investigated in the subsequent “Mathematical model” section. In the next “The Chebyshev polynomials of the second kind: shifted version on [*t*_*a*_, *t*_*b*_]” section we first give a review of the SCPSK functions. Also, a rigorous mathematical proof is given for the convergence analysis of the SCPSK bases. “QLM-SCPSK collocation strategy based on splitting of time interval” section first describes the fundamental ideas of the QLM. Hence, the methodology of the spectral domain decomposition approach is illustrated and finally, the basic steps of the proposed QLM-SCPSK algorithm are given in detail. In “Experimental calculations” section some numerical simulations on a long-time domain are carried out to support the theoretical findings. The last “Conclusions” section includes a summary of the presented research study.

## Mathematical model

A dynamical model that is introduced in this paper is based on the Kermak and Mckendrick model [3]. The model is introduced for the omicron version of Coronavirus. During the outbreak of the omicron, a large number of people in the community had received the vaccine. Therefore, the population of susceptible, infected, recovered, and isolated are divided into two groups, vaccinated and unvaccinated. Consider, *S*(*t*), $S_v(t)$ be the populations of unvaccinated and vaccinated susceptible at time *t* respectively. Denote further by *I*(*t*) and $I_v(t)$ as the number of unvaccinated and vaccinated infected people. Also, *R*(*t*) and $R_v(t)$ are unvaccinated recovered and vaccinated recovered persons. Finally, *J*(*t*) and $J_v(t)$ are the number of unvaccinated and vaccinated isolated people.

We consider The fraction $\beta$ of susceptible people who get infected. This fraction of unvaccinated is $\beta '$. Also, a fraction $\delta$ of vaccinated susceptible goes to $R_v$ class without infection. The recovery rates are $\gamma _1$ and $\gamma _2$ for unvaccinated and vaccinated infected people. The unvaccinated and vaccinated infected people are isolated with rates $\alpha _1$ and $\alpha _2$ respectively. The recovery rate in *J* and $J_v$ groups are $\eta _1$ and $\eta _2$. Two parameters $\mu _1$ and $\mu _2$ are mortality rates because of disease in unvaccinated and vaccinated isolated populations. In the end, unvaccinated and vaccinated recovered people are reinfected with the rate of $\theta _1$ and $\theta _2$. In this model, $\Lambda$ is the birth rate of the population. The dynamical model is given by

1 $$\begin{aligned} \left\{ \begin{array}{l} \dot{S} = \Lambda - \beta S(I+I_v)- (\lambda +\mu ) S+ \theta _1 R,\\ \dot{S_v}= -\beta ' S_v(I+ I_v)+ \theta _2 R_v+ \lambda S- (\delta +\mu ) S_v,\\ \dot{I} = \beta S(I+ I_v)- (\gamma _1+\alpha _1+\mu ) I, \\ \dot{I_v}= \beta ' S_v(I+ I_v)- (\gamma _2+\alpha _2+\mu )I_v,\\ \dot{R} = \gamma _1 I-(\theta _1+\mu ) R+ \eta _1 J, \\ \dot{R_v}= \gamma _2 I_v- (\theta _2+\mu ) R_v+ \eta _2J_v+ \delta S_v,\\ \dot{J} = \alpha _1 I- (\eta _1+\mu _1)J,\\ \dot{J_v}= \alpha _2I_v- (\eta _2+\mu _2) J_v. \end{array}\right. \end{aligned}$$

**Fig. 1:**
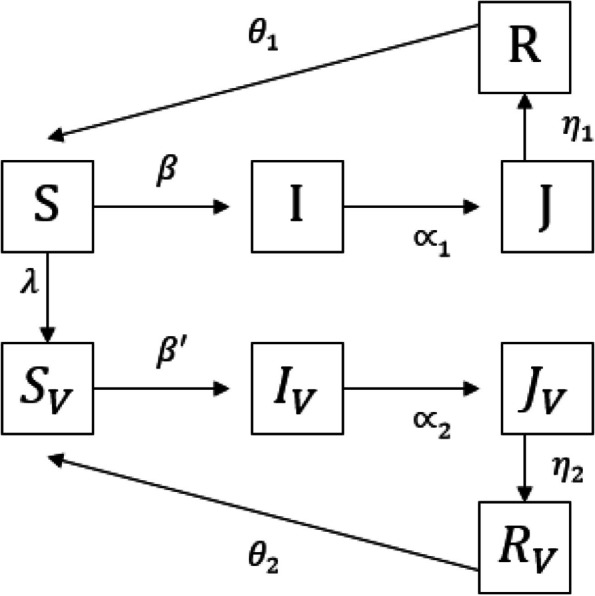
Schematic view of the COVID model

The flowchart of the model is given in Fig. 1.

The above model is subjected to the following initial conditions

2 $$\begin{aligned} \left\{ \begin{array}{l} S(0)=S_0,\quad ~ S_v(0)=S_{v0}, \quad ~ I(0)=I_0,\quad ~ I_v(0)=I_{v0}, \\ R(0)=R_0,\quad R_v(0)=R_{v0},\quad J(0)=J_0,\quad J_v(0)=J_{v0}. \end{array}\right. \end{aligned}$$

Let us derive the equilibrium points of the given model Eq. (1). The first equilibrium point is the disease-free one. It is given by

3 $$\begin{aligned} E_0:=\left( S^0, S_v^0, I^0, I_v^0, R^0, R_v^0, J^0, J_v^0\right) =\left(\frac{\Lambda }{\lambda +\mu }, \frac{\Lambda }{\delta +\mu }, 0, 0, 0, 0, 0, 0\right). \end{aligned}$$

The other equilibrium point is obtained as follows

4 $$\begin{aligned} E^*:=(S^*, S_v^*, I^*, I_v^*, R^*, R_v^*, J^*, J_v^*), \end{aligned}$$

where

$$\begin{aligned} S^*{} & {} = \frac{\Lambda + x_1 I^*}{\beta (I^*+I_v^*)+ \lambda +\mu },\\ S_v^*{} & {} = \frac{1}{\beta (I^*+ I_v^*)}\left( x_2 I_v^*+ \frac{(\lambda +\mu )(\Lambda + x_1I^*)}{\beta (I^*+I_v^*)+ \lambda +\mu }\right) ,\\ I_v^*{} & {} = \frac{1}{\beta } (\frac{(\alpha _1+ \gamma _1+ \mu )(1+ \lambda + \mu )}{\Lambda + x_1 I^*}- \beta )I^*,\\ R^*{} & {} = \frac{\gamma _1 I^*+ \eta _1 J^*}{\theta _1},\quad R_v^*= \frac{\gamma _2I_v^*+ \eta _2 J_v^*+ (\delta +\mu ) S_v^*}{\theta _2},\\ J^*{} & {} =\frac{\alpha _1 I^*}{\eta _1+ \mu _1},\quad J_v^*= \frac{\alpha _2 I_v^*}{\eta _2+ \mu _2}. \end{aligned}$$

Also, we have defined two parameters $x_1$ and $x_2$ as

$$\begin{aligned} x_1= \gamma _1+ \frac{\alpha _1 \eta _1}{\mu _1 + \eta _1}, \quad x_2= \gamma _2+ \frac{\alpha _2 \eta _2}{\mu _2 + \eta _2}. \end{aligned}$$

By doing some calculations we find that $I^*$ satisfies in the following algebraic equation

5 $$\begin{aligned} a_2\,I^{*2}+ a_1\,I^*+a_0=0, \end{aligned}$$

where the coefficients $a_i$, for $i=0,1,2$ are given by

$$\begin{aligned} a_0{} & {} = y_4\Lambda ^2- y_5y_6\beta \Lambda (y_1- \beta \Lambda ),\\ a_1{} & {} = \frac{1}{\beta }y_1y_2y_3(y_1- \beta \Lambda )+ 2x_1y_4\Lambda - y_5(y_1- \beta \Lambda )\left(\frac{\beta 'y_1}{\beta }+ \beta x_1y_6\right)+ \beta ^2x_1y_5y_6\Lambda ,\\ a_2{} & {} = x_1^2y_4- x_1y_1y_2y_3+ \beta x_1\left(\frac{\beta ' y_1}{\beta }+ \beta x_1y_6\right). \end{aligned}$$

Also, we have

$$\begin{aligned} y_1{} & {} = (\alpha _1+ \gamma _1+ \mu )(1+ \lambda + \mu ),~~~~~~~y_2=\frac{\beta ' \theta _2}{\theta _2+ \mu },~~~~ y_3=\gamma _2+ \frac{\eta _2\alpha _2}{\eta _2+ \mu _2},\\ y_4{} & {} = \lambda \beta '(\alpha _1+ \gamma _1 + \mu ),~~~~~~y_5= \alpha _2+ \gamma _2 + \mu ,~~~~~~y_6= \delta + \mu + \frac{\delta \theta _2}{\theta _2+ \mu }. \end{aligned}$$

If $y_1>\beta \Lambda$, the Eq. 5 has a positive root. Hence, the endemic equilibrium point $E^*$ exists.

###  Boundedness of system

Our goal here is to examine the boundedness property of the COVID-19 system. To proceed, we have

#### Theorem 2.1

All solutions of the COVID-19 system Eq. (1) are uniformly bounded.

#### Proof

Consider the new variable

$$\begin{aligned} X(t)= S(t)+ S_v(t)+ I(t)+ I_v(t)+ R(t)+ R_v(t)+ J(t)+ J_v(t). \end{aligned}$$

Thus, we have

$$\begin{aligned} \frac{dX}{dt}= \frac{dS}{dt}+ \frac{dS_v}{dt}+ \frac{dI}{dt}+ \frac{dI_v}{dt}+ \frac{dR}{dt}+ \frac{dR_v}{dt}+ \frac{dJ}{dt}+ \frac{dJ_v}{dt}. \end{aligned}$$

Since, $\mu < \mu _i$ for $i=1, 2$, we arrive at

$$\begin{aligned} \frac{dX}{dt}= \Lambda - \mu (S+ S_v+ I+ I_v+ R+ R_v)- \mu _1 J- \mu _2 J_v\le \Lambda - \mu X. \end{aligned}$$

By solving the above linear differential equation in terms of variable *X* one gets,

$$\begin{aligned} X(t)\le \frac{\Lambda }{\mu }+\left(X_0- \frac{\Lambda }{\mu }\right) e^{-\mu t}. \end{aligned}$$

Now, by tending $t\rightarrow \infty$, we obtain $0\le X\le \frac{\Lambda }{\mu }$. Therefore, all solutions of the system, i.e., $(S, S_v, I, I_v, R, R_v, J, J_v)$ of the system are confined the region

$$\begin{aligned} W:=\left\{ X\in \mathbb {R}^8~:~0\le X<\frac{\Lambda }{\mu }+\epsilon \right\} , \end{aligned}$$

for any $\epsilon >0$ as $t\rightarrow \infty$. $\square$

### Basic reproduction number

Let us present the basic reproduction number by $R_0$. It is the most important epidemiological parameter. It is defined as the “number of secondary infected individuals caused by a single infected individual in the whole time interval” [34]. In this paper, using the method presented in the paper [34], we calculate $R_0$ value. The compartments that include infected people are $I, I_v, J, J_v$. Therefore, we form the following system

$$\begin{aligned} \left\{ \begin{array}{l} \dot{I}= \beta S(I+ I_v)- \gamma _1 I- \alpha _1 I- \mu I, \\ \dot{I_v}= \beta ' S_v(I+ I_v)- \gamma _2 I_v- \alpha _2I_v- \mu I_v,\\ \dot{J}= \alpha _1 I- \eta _1 J- \mu _1 J,\\ \dot{J_v}= \alpha _2I_v- \eta _2 J_v- \mu _2 J_v. \end{array}\right. \end{aligned}$$

The above system can be rewritten as

$$\begin{aligned} \frac{dy}{dt}= \varphi (y)-\psi (y), \end{aligned}$$

where, $$y=(I, I_v, J, J_v)^T$$, and

$$\begin{aligned} \varphi (y){} & {} =\left( \beta S(I+ I_v), \beta ' S_v(I+ I_v), 0, 0\right) ^T,\\ \psi (y){} & {} = \left( -(\gamma _1+\alpha _1+ \mu )I, -(\gamma _2+ \alpha _2+ \mu )I_v, \alpha _1I- (\eta _1+ \mu _1)J, (\alpha _2-\eta _2- \mu _2)J_v)\right) ^T. \end{aligned}$$

Now, let us obtain the Jacobian matrix related to $\varphi$ and $\psi$ at $E_0$ as the disease free equilibrium point. We have

$$\begin{aligned} F= \left[ \begin{array}{cccc} \beta S^0 &{} \beta S^0 &{} 0 &{} 0 \\ \beta ' S_v^0&{} \beta ' S_v^0 &{} 0 &{} 0 \\ 0 &{} 0 &{} 0 &{} 0 \\ 0 &{} 0 &{} 0 &{} 0\\ \end{array}\right] , \end{aligned}$$

and

$$\begin{aligned} V= \left[ \begin{array}{cccc} -(\gamma _1+\alpha _1+ \mu ) &{} 0 &{} 0 &{} 0 \\ 0&{} -(\gamma _2+ \alpha _2+ \mu ) &{} 0 &{} 0 \\ \alpha _1&{} 0 &{} - (\eta _1+ \mu _1) &{} 0 \\ 0 &{} 0 &{} 0 &{} \alpha _2-\eta _2- \mu _2 \\ \end{array}\right] . \end{aligned}$$

As we know that the basic number, $R_0$, is the eigenvalue of the matrix $FV^{-1}$. It is given by some simple calculations as

6 $$\begin{aligned} R_0= \frac{\beta S^0}{\gamma _1+\alpha _1+ \mu } + \frac{\beta ' S_v^0}{\gamma _2+ \alpha _2+ \mu }. \end{aligned}$$

#### Theorem 2.2

Under the restriction $R_0<1$, the point $E_0$ (disease free equilibrium point) is asymptotically stable.

#### Proof

Denote the Jacobian matrix of COVID-19 system Eq. (1) by $\varvec{J}:=\varvec{J}(S,S_v,I,I_v)$. It has the form

7 $$\begin{aligned} \varvec{J}= \left[ \begin{array}{cccccccc} -\beta \,I_1-\lambda -\mu &{}0 &{} -\beta \,S &{}-\beta \, S &{}\theta _1 &{}0 &{}0 &{}0\\ \lambda &{}-\beta '\,I_1-\delta -\mu &{} -\beta '\,S_v &{}-\beta '\,S_v &{}0 &{}\theta _2 &{}0 &{}0\\ \beta \,I_1 &{}0 &{} \beta \,S-\zeta _1 &{}\beta \, S &{}0 &{}0 &{}0 &{}0\\ 0 &{}-\beta '\,I_1 &{} -\beta '\,S_v &{}-\beta '\,S_v-\zeta _2&{}0 &{}0 &{}0 &{}0\\ 0 &{}0 &{}\gamma _1 &{}0 &{}-\theta _1-\mu &{}0 &{}\eta _1&{}0\\ 0 &{}\delta &{}0 &{}\gamma _2 &{}0 &{}-\theta _2-\mu &{}0 &{}\eta _2\\ 0 &{}0 &{}\alpha _1 &{}0 &{}0 &{}0 &{}-\eta _1-\mu _1&{}0\\ 0 &{}0 &{}0 &{}\alpha _2 &{}0 &{}0 &{}0 &{}-\eta _2-\mu _2\\ \end{array}\right] , \end{aligned}$$

where $I_1:=I+I_v$ and $\zeta _j:=\gamma _j+\alpha _j+\mu$ for $j=1,2$. The former Jacobian matrix at the equilibrium point $E_0$ can be written as

$$\begin{aligned} \varvec{J}(E_0):= \left[ \begin{array}{cccccccc} -\lambda -\mu &{}0 &{} -\beta S^0 &{}-\beta S^0 &{}\theta _1 &{}0 &{}0 &{}0\\ \lambda &{}-\delta -\mu &{} -\beta ' S^0_v &{}-\beta ' S^0_v &{}0 &{}\theta _2 &{}0 &{}0\\ 0 &{}0 &{} \beta S^0-\zeta _1 &{}\beta S^0 &{}0 &{}0 &{}0 &{}0\\ 0 &{}0 &{} \beta 'S^0_v &{}\beta ' S^0_v-\zeta _2 &{}0 &{}0 &{}0 &{}0\\ 0 &{}0 &{}\gamma _1 &{}0 &{}-\theta _1-\mu &{}0 &{}\eta _1&{}0\\ 0 &{}\delta &{}0 &{}\gamma _2 &{}0 &{}-\theta _2- \mu &{}0 &{}\eta _2\\ 0 &{}0 &{}\alpha _1 &{}0 &{}0 &{}0 &{}-\eta _1-\mu _1&{}0\\ 0 &{}0 &{}0 &{}\alpha _2 &{}0 &{}0 &{}0 &{}-\eta _2-\mu _2\\ \end{array}\right] . \end{aligned}$$

The characteristic equation is $p(x):=\det (\varvec{J}(E_0)-x\varvec{I})=0$. By computing the determinant and after some manipulations we get

8 $$\begin{aligned} p(x)=-(\lambda +\mu +x)q_1(x)\left\{ p_1(x)\,p_2(x)- \beta \beta ' S^0 S^0_v\right\} \Big \{(\delta +\mu +x)\left[ q_2(x)+\delta \theta _2(\theta _2+\mu +x)\right] \Big \}, \end{aligned}$$

where we have

$$\begin{aligned} q_j(x){} & {} :=(\theta _j +\mu + x)(\mu _j+ \eta _j+x),\quad j=1,2,\\ p_1(x){} & {} :=\beta S^0-\zeta _1-x,\\ p_2(x){} & {} :=\beta ' S^0_v-\zeta _2-x. \end{aligned}$$

Owing to the fact that we have $R_0<1$, one can easily check that all roots are negative. Hence, the equilibrium point $E_0$ is asymptotically stable. $\square$

### Bifurcation

In this section, we describe the existence of bifurcation at threshold number $R_0=1$. We select $\beta$ to be the bifurcation coefficient that yields bifurcation at $R_0=1$. The model takes the form

$$\begin{aligned} \left\{ \begin{array}{l} f_1 = \Lambda - \beta S(I+I_v)- (\lambda +\mu ) S+ \theta _1 R,\\ f_2= -\beta ' S_v(I+ I_v)+ \theta _2 R_v+ \lambda S- (\delta +\mu ) S_v,\\ f_3 = \beta S(I+ I_v)- (\gamma _1+\alpha _1+\mu ) I, \\ f_4= \beta ' S_v(I+ I_v)- (\gamma _2+\alpha _2+\mu )I_v,\\ f_5 = \gamma _1 I-(\theta _1+\mu ) R+ \eta _1 J, \\ f_6= \gamma _2 I_v- (\theta _2+\mu ) R_v+ \eta _2J_v+ \delta S_v,\\ f_7 = \alpha _1 I- (\eta _1+\mu _1)J,\\ f_8= \alpha _2I_v- (\eta _2+\mu _2) J_v. \end{array}\right. \end{aligned}$$

Here, the transmission rate, $\beta '$, is less than $\beta$. According to the available information, consider $\beta '= \frac{\beta }{30}$. Hence, $R_0=1$ gives

$$\begin{aligned} \beta =\frac{30\xi _1\xi _2}{30S^0\xi _2+ S_v^0\xi _1}=\beta ^*. \end{aligned}$$

Next, we construct characteristic polynomial at $E_0$ and bifurcation parameter $\beta ^*$ as follows

$$\begin{aligned} p(x)=-(\lambda + \mu + x)q_1(x)\left( x^2-x\left( \frac{\xi _1\xi _2\,(30S^0+ S^0_v)}{30S^0\xi _2+ S_v^0\xi _1}- \xi _1- \xi _2\right) \right) \Big \{(\delta + \mu + x)\big [q_2(x)+ \delta \theta _2(\theta _2+ \mu + x)\big ]\Big \}. \end{aligned}$$

where $q_1(x)$ and $q_2(x)$ are defined in Eq. (8). We can easily verify that all roots are negative except one of them is zero. Therefore, there exists bifurcation at $R_0=1$.

Now, we compute the right eigenvector $w=(w_1, w_2, w_3, w_4, w_5, w_6, w_7, w_8)^T$. Corresponding to the zero eigenvalue that satisfies $J(E_0)w=0$.

By solving the equation, we obtain

$$\begin{aligned} w_4{} & {} =\frac{\xi _1- \beta S^0}{\beta S^0}w_3,~~\qquad \qquad \qquad \qquad \quad \, w_7=\frac{\alpha _1}{\eta _1 + \mu _1}w_3,\\ w_8{} & {} =\frac{-\alpha _2}{\mu _2+ \eta _2}w_4,~~~~\qquad \qquad \qquad \qquad \quad \, w_5= \frac{1}{\theta _1+ \mu }(\gamma _1 w_3+ \eta _1 w_4),\\ w_1{} & {} = \frac{1}{\lambda + \mu }(\theta _1 w_5- \beta S^0(w_3- w_4),\quad w_2= \frac{\theta _2(\gamma _2 w_4- \eta _2 w_4)}{(\theta _2+ \mu )(\delta + \mu )- \theta _2 \delta },\\ w_6{} & {} =\frac{1}{\theta _2}(\beta 'S_v^0(w_3+ w_4)+ (\delta + \mu )w_3- \lambda w_1). \end{aligned}$$

To compute the left eigenvector $V=(v_1, v_2, v_3, v_4, v_5, v_6, v_7, v_8)$, we solve the equation $V\cdot J(E_0)=\textbf{0}$. We obtain $v_1=0, v_2=0, v_4=\frac{\xi _1- \beta S^0}{\beta ' S_v^0}v_3,v_5= v_6= v_7= v_8=0.$

Using Theorem 4.1 in [35], we compute the bifurcation coefficients *a* and *b* as follow. For the first one, we have

$$\begin{aligned} a{} & {} = \sum \limits _{i,j=1}^8v_3w_iw_j\frac{\partial ^2f_3}{\partial x_i \partial x_j}(E_0, \beta ^*)+ \sum \limits _{i,j=1}^8v_4w_iw_j\frac{\partial ^2f_4}{\partial x_i \partial x_j}(E_0, \beta ^*)\\{} & {} = 2w_3(v_3w_1\beta + w_2v_4\beta ')= \frac{2\xi _1}{S^0}(w_1+ x_1w_2)v_3, \end{aligned}$$

where, $x_1= \frac{\xi _1- \beta S^0}{\beta S_v^0}$. One can be easily seen that $a>0$.

Similarly, for the second parameter *b*, we have

$$\begin{aligned} b=\sum \limits _{k, i=1}^8v_kw_i\frac{\partial ^2 f_k}{\partial x_i\partial \beta }(E_0, \beta ^*)=(w_3+ w_4)\left(v_3S^0+ \frac{1}{30}v_4S_v^0\right)= \frac{\xi _1^2}{\beta }v_3w_3>0. \end{aligned}$$

Thus, if $\beta =\beta ^*$, the disease-free equilibrium point $E_0$ is unstable. If $R_0<1$, $E_0$ is locally asymptotically stable. Otherwise, $E_0$ is unstable.

## The Chebyshev polynomials of the second kind: shifted version on [*t*_*a*_, *t*_*b*_]

In order to devise our multi-domain collocation scheme, we need to introduce the set of (orthogonal) polynomials known as the Chebyshev polynomials of the second kind (CPSK), see [36]. These polynomials on $[-1,1]$ are defined by the formula

9 $$\begin{aligned} U_j(p):=\sin \left[ (j+1)\Theta \right] /\sin \Theta ,\quad p=\cos \Theta , \end{aligned}$$

where $\Theta \in [0,\pi ]$ and $j\ge 0$. The set of CPSK polynomials constitutes an orthogonal system on $[-1,1]$ with regard to positive function $w_1(p)=(1-p^2)^{\frac{1}{2}}$. The shifted version of them are obtained by using $p=(2z-1)$ on [0, 1]. They have the explicit representation as [37]

10 $$\begin{aligned} U_j(z)=\sum \limits _{i=0}^{j}(-1)^{j-i}\,4^{i}\frac{(j+i+1)!}{(j-i)!\,(2i+1)!}\,z^{i},\quad j>0, \end{aligned}$$

with $U_0(z)=1$. The second one is obtained as $U_1(z)=4z-2$. In addition to Eq. (10), we may use the next recursive formula to get the remaining polynomials as

11 $$\begin{aligned} U_{j+1}(z)=(4z-2)\,U_{j}(z)-U_{j-1}(z),\quad j\ge 1. \end{aligned}$$

Moreover, the orthogonality condition of these shifted polynomials with regard to weight function $w_2(z)=(z-z^2)^{\frac{1}{2}}$ is stated as

12 $$\begin{aligned} \int _{0}^1 U_j(z)\,U_{r}(z)\,w_2(z)dz=\frac{\pi }{8} \left\{ \begin{array}{ll} 0, &{} j\ne r,\\ 1, &{} j=r. \end{array}\right. \end{aligned}$$

We finally notice that the roots of $U_j(z)$ are located inside the interval (0, 1). Precisely speaking, these zeros are [37]

13 $$\begin{aligned} z_{m}=\frac{1}{2}\left( 1+\cos \left( \frac{m\,\pi }{j+1}\right) \right) ,\quad m=1,2,\ldots ,j. \end{aligned}$$

We will utilize the shifted CPSK on an arbitrary interval $[t_a,t_b]$. Toward this end, we take $z=(t-t_a)/T$ or $t=T z+t_a$, where $T=t_b-t_a$. So, we have

### Definition 3.1

The shifted CPSK (SCPSK) on $[t_a,t_b]$ is defined via relation

14 $$\begin{aligned} \mathbb {U}_j(t)=U_j\left( \frac{t-t_a}{T}\right) ,\quad t\in [t_a,t_b]. \end{aligned}$$

From the above change of variable, we are able to represent the summation Eq. (10) as

15 $$\begin{aligned} \mathbb {U}_j(t)=\sum \limits _{i=0}^{j} o_{ji}\,(t-t_a)^{i},\quad j>0, \end{aligned}$$

where

$$\begin{aligned} o_{ji}:=(-1)^{j-i}\,\left( \frac{4}{T}\right) ^{i}\frac{(j+i+1)!}{(j-i)!\,(2i+1)!}. \end{aligned}$$

With the preceding transformation, the orthogonality condition Eq. (12) is changed accordingly. The orthogonality of the set $\{\mathbb {U}_j(t)\}_{j=0}^{\infty }$ will be gotten with regard to the weight function $w_a(t)=\sqrt{T(t-t_a)-(t-t_a)^2}$. We have indeed the next formula

16 $$\begin{aligned} \int _{t_a}^{t_b} \mathbb {U}_j(t)\,\mathbb {U}_{r}(t)\,w_a(t)dt=\frac{T^2\,\pi }{8} \left\{ \begin{array}{ll} 0, &{} j\ne r,\\ 1, &{} j=r. \end{array}\right. \end{aligned}$$

Finally, based on relations $t=T z+t_a$ and Eq. (13) we can locate the zeros of SCPSK $\mathbb {U}_j(t)$ on $(t_a,t_b)$. The associated zeros are

17 $$\begin{aligned} t_{m}=\frac{1}{2}\left( t_a+t_b+T\,\cos \left( \frac{m\,\pi }{j+1}\right) \right) ,\quad m=1,2,\ldots ,j. \end{aligned}$$

These finite number of points will be utilized as the collocation nodes in the main proposed algorithm, below.

### Function approximation: convergence and error analysis of SCPSK

To continue, let us suppose that a given function *x*(*t*) is belonged to the weighted $L^2$ space on $[t_a,t_b]$. It then can be expressed as a summation of SCPSK in the form

18 $$\begin{aligned} x(t)=\sum \limits _{j=0}^{\infty } \nu _j\,\mathbb {U}_j(t),\quad t\in [t_a,t_b]. \end{aligned}$$

To obtain the unknown coefficients $\nu _j,j\ge 0$, the aim is to exploit the orthogonality property Eq. (16). It gives us

19 $$\begin{aligned} \nu _j:=\frac{2}{T^2\,\pi }\int _{t_a}^{t_b} \mathbb {U}_{j}(t)\,x(t)\,w_a(t)\,dt,\quad j=1,2,\ldots . \end{aligned}$$

We first find an upper bound for the coefficients $\nu _j$ in terms of *j*. Hence, we show that series form Eq. (18) is convergent uniformly on $[t_a,t_b]$. To continue. let assume that $M_{ab}:=\max _{t\in [t_a,t_b]}\left| x''(t)\right|$. Moreover, the space $L^2_w$ stands for the space of square-integrable functions on $[t_a,t_b]$ with regard to weight function $w_a(t)$ defined previously. Thus, we have the next assertion.

#### Theorem 3.1

For the function $x(t)\in C^{(2)}([t_a,t_b])\cap L^2_w([t_a,t_b])$, which can be written as Eq. (18) we obtain the following estimate

20 $$\begin{aligned} \left| \nu _j\right| < \left( \frac{32\,T^2\,M_{ab}}{\pi }\right) \,\frac{1}{j^5},\quad j>1. \end{aligned}$$

#### Proof

We begin by the relation Eq. (19) and make the change of variable $t-t_a=\frac{T}{2}(1+\cos \Theta )=:r(\Theta )$ we arrive at

21 $$\begin{aligned} \nu _j=\frac{2}{\pi }\int _0^{\pi } \sin [(j+1)\Theta ]\,x\left( r(\Theta )\right) \,\sin \Theta \,d\Theta = \frac{1}{\pi }\int _0^{\pi } x\left( r(\Theta )\right) \,\left( \cos [j\Theta ]-\cos [(j+2)\Theta ]\right) \,d\Theta . \end{aligned}$$

We then integrate by parts two times on the last formula. By introducing the auxiliary function

$$\begin{aligned} H_j(\Theta ):=\frac{1}{j}\left( \frac{\sin ((j-1)\Theta )}{j-1}-\frac{\sin ((j+1)\Theta )}{j+1}\right) -\frac{1}{j+2}\left( \frac{\sin ((j+1)\Theta )}{j+1}-\frac{\sin ((j+3)\Theta )}{j+3}\right) , \end{aligned}$$

we get from Eq. (21) the next expression for $\nu _j$ as

22 $$\begin{aligned} \nu _j=\frac{T^2}{8\pi }\int _0^{\pi } x''\left( r(\Theta )\right) \,H_j(\Theta )\,\sin (\Theta )\,d\Theta . \end{aligned}$$

We next utilize the fact that $\left| x''\left( r(\Theta )\right) \right| \le M_{ab}$ and $|\sin (\Theta )|\le 1$. The resulting inequality is

23 $$\begin{aligned} \left| \nu _j\right| \le \frac{T^2\,M_{ab}}{8\pi }\left| \int _0^{\pi } H_j(\Theta )\,d\Theta \right| . \end{aligned}$$

A simple calculation can be done to obtain the exact values of the integral on the former line. It follows that

$$\begin{aligned} \int _0^{\pi } H_j(\Theta )d\Theta =\frac{1-(-1)^{j-1}}{(j-1)^2j}+\frac{(-1)^{j+1}-1}{j(j+1)^2}+\frac{(-1)^{j+1}-1}{(j+1)^2(j+2)}+\frac{1-(-1)^{j+3}}{(j+2)(j+3)^2}. \end{aligned}$$

By utilizing an odd $j>1$, the resulting integral’s value is zero obviously. If we choose a $j>1$ to be even, then we have

$$\begin{aligned} \int _0^{\pi } H_j(\Theta )\,d\Theta = \frac{2}{j}\left[ \frac{1}{(j-1)^2}-\frac{1}{(j+1)^2}\right] +\frac{2}{j+2}\left[ \frac{1}{(j+3)^2}-\frac{1}{(j+1)^2}\right] = \frac{64/(j+1)}{(j-1)^2(j+3)^2}. \end{aligned}$$

We use the relation $j-1\ge \frac{j}{2}$, which holds for all $j\ge 2$. Hence, we reach at the inequality

24 $$\begin{aligned} \left| \int _0^{\pi } H_j(\Theta )\,d\Theta \right| < \frac{256}{j^5}. \end{aligned}$$

By placing Eq. (24) into Eq. (23) yields the result Eq. (20).

To handle the model problem Eq. (1) in practical situations, we require to truncate the infinite series solution Eq. (18) into a finite one. If we use only $(\mathcal {J}+1)$ ($\mathcal {J}\in \mathbb {N}$) basis functions, we can approximate *x*(*t*) in the way that

25 $$\begin{aligned} x(t)\approx x_{\mathcal {J}}(t):=\sum \limits _{j=0}^{\mathcal {J}} \nu _j\,\mathbb {U}_j(t),\quad t\in [t_a,t_b]. \end{aligned}$$

So, our goal now is to examine the difference between *x*(*t*) and $x_{\mathcal {J}}(t)$ as an approximation for it. This global error is defined by the formula

26 $$\begin{aligned} \mathbb {E}_{\mathcal {J}}(t):=x(t)-x_{\mathcal {J}}(t)=\sum \limits _{j=\mathcal {J}+1}^{\infty } \nu _j\,\mathbb {U}_j(t). \end{aligned}$$

To get an upper bound for the global error $\mathbb {E}_{\mathcal {J}}(t)$, we employ the following relation is valid for the normal CPSK on $[-1,1]$ given as

$$\begin{aligned} \left| U_j(p)\right| \le j+1,\quad \forall p\in [-1,1]. \end{aligned}$$

With the aid of transformation $p=2(t-t_a)/T-1$, we arrive at the same conclusion for $\mathbb {U}_j(t)$ on $[t_a,t_b]$. Namely, we have

27 $$\begin{aligned} \left| \mathbb {U}_j(t)\right| \le j+1\le 2j,\quad \forall j\in \mathbb {N},~\forall t\in [t_a,t_b]. \end{aligned}$$

The next result establishes that the norm of error $\Vert \mathbb {E}_{\mathcal {J}}\Vert _{\infty }$ converges to 0 if we let $\mathcal {J}$ goes to infinity.

#### Theorem 3.2

Under assumptions of Theorem 3.1, the error $\mathbb {E}_{\mathcal {J}}(t)$ converges to 0 as $\mathcal {J}\rightarrow \infty$. In fact, we have

$$\begin{aligned} \Vert \mathbb {E}_\mathcal {J}\Vert _{\infty }\le \left( \frac{64\,T^2\,M_{ab}}{3\pi } \right) \frac{1}{\mathcal {J}^3}. \end{aligned}$$

#### Proof

To prove this result, we consider Eq. (26) together with Eq. (27) to obtain

$$\begin{aligned} \left| E_\mathcal {J}(t)\right| \le \sum \limits _{j=\mathcal {J}+1}^{\infty }\left| \nu _j\right| \,\left| \mathbb {U}_j(t)\right| \le 2\sum \limits _{j=\mathcal {J}+1}^{\infty }j\,\left| \nu _j\right| . \end{aligned}$$

Hence, we apply the upper bound for $\left| \nu _j\right|$ derived in Eq. (20). The resultant inequality is

28 $$\begin{aligned} \left| E_\mathcal {J}(t)\right| < L\sum \limits _{j=\mathcal {J}+1}^{\infty }\frac{1}{j^4}, \quad L:=\frac{64\,T^2\,M_{ab}}{\pi }. \end{aligned}$$

Utilizing the well-known Integral Test [38] gives us

$$\begin{aligned} \sum \limits _{j=\mathcal {J}+1}^{\infty } \frac{1}{j^4}\le \int _\mathcal {J}^{\infty } \frac{dy}{y^4}=\frac{1}{3\mathcal {J}^3}. \end{aligned}$$

Our proof is established by inserting the foregoing result into Eq. (28) followed by taking the supremum over all $t\in [t_a,t_b]$. By tending $\mathcal {J}$ to infinity, we have done the proof.

## QLM-SCPSK collocation strategy based on splitting of time interval

Let us emphasize that the spectral matrix collocation approach based on the SCPSK may not yield convergence on a long time interval $[t_a,t_b]$. One remedy is to use a large number of bases on the long domains accordingly to reach the desired level of accuracy. Another approach is to divide the given interval into a sequence of subintervals and employ the proposed collocation scheme on each subinterval consequently.

Towards this end, we split the time interval $[t_a,t_b]$ into $N\ge 1$ subdomains in the forms

$$\begin{aligned} K_n:=[t_{n},t_{n+1}],\quad n=0,1,\ldots , N-1. \end{aligned}$$

Here, we have $t_0:=t_a$ and $t_N:=t_b$. The uniform time step is taken as $h=t_{n+1}-t_n=(t_b-t_a)/N$. Note that by selecting $N=1$, we turn back to the traditional spectral collocation method on the whole domain $[t_a,t_b]$. Therefore, on each subinterval $K_n$ we take the approximate solution of the model Eq. (1) to be in the form Eq. (25) as

29 $$\begin{aligned} x^n_{\mathcal {J}}(t):=\sum \limits _{j=0}^{\mathcal {J}} \omega ^n_j\,\mathbb {U}_j(t)=\varvec{U}_{\mathcal {J}}(t)\,\varvec{W}^n_\mathcal {J},\quad t\in K_n, \end{aligned}$$

where we utilized the notations

$$\begin{aligned} \varvec{W}_{\mathcal {J}}^n:=\left[ \omega ^n_0\quad \omega ^n_1\quad \ldots \quad \omega ^n_{\mathcal {J}}\right] ^T,\quad \varvec{U}_{\mathcal {J}}(t):=\left[ \mathbb {U}_0(t)\quad \mathbb {U}_1(t)\quad \ldots \quad \mathbb {U}_J(t)\right] , \end{aligned}$$

as the vector of unknown coefficients and the vector of SCPSK bases respectively. Once we get the all local approximate solutions for $n=0,1,\ldots ,N-1$, the global approximate solution on the given (large) interval $[t_a,t_b]$ will be constructed in the form

$$\begin{aligned} x_{\mathcal {J}}(t)=\sum \limits _{n=0}^{N-1} c_n(t)\,x^n_{\mathcal {J}}(t),\quad c_n(t):= \left\{ \begin{array}{ll} 0, &{} t\notin K_n,\\ 1, &{} t\in K_n.\\ \end{array}\right. \end{aligned}$$

In order to collocate a set of $(\mathcal {J}+1)$ linear equations to be obtained later at some suitable points, we consider the roots of $\mathbb {U}_{\mathcal {J}+1}(t)$ on the subinterval $K_n$. By modifying the points given in Eq. (17), we take the collocation nodes as

30 $$\begin{aligned} t_{\nu ,n}=\frac{1}{2}\left( t_n+t_{n+1}+h\,\cos \left( \frac{\nu \,\pi }{\mathcal {J}+2}\right) \right) ,\quad \nu =1,2,\ldots ,\mathcal {J}+1. \end{aligned}$$

At the end, we note that in the proposed splitting approach, the given initial conditions of the underlying model problem are prescribed on the first subinterval $K_0$. Once the approximate solution on $K_0=[t_0,t_1]$ is determined, we utilize it to assign the initial conditions on the next time interval $K_1$. To do so, it is sufficient to evaluate the obtained approximation at $t_1$. We repeat this idea on the next subintervals in order until we arrive at the last subinterval $K_{N-1}$. Below, we illustrate the main steps of our matrix collocation algorithm on an arbitrary subinterval $K_n$ for $n=0,1,\ldots ,N-1$.

### The QLM-SCPSK matrix collocation technique

Our chief aim is to solve the nonlinear COVID-19 system Eq. (1) efficiently by using the spectral method based on SCPSK basis. Towards this end, we first need to get rid of the nonlinearity of the model. This can be done by employing the Bellman’s quasilinearization method (QLM) [39]. Thus we will get more advantages in terms of running time, especially for large values of *J* in comparison to the performance of directly applied collocation methods to nonlinear models, see cf. [40–42]. By combining the idea of QLM and the splitting of the domain we will obtain more gains in terms of accuracy for the approximate solutions of nonlinear model Eq. (1). Let us first describe the technique of QLM. For more information, we may refer the readers to the above-mentioned works.

By reformulating the original COVID-19 model Eq. (1) in a compact form we get

31 $$\begin{aligned} \frac{d}{dt} \varvec{z}(t)=\varvec{G}(t,\varvec{z}(t)), \end{aligned}$$

where

$$\begin{aligned} \varvec{z}(t)=\left[ \begin{array}{c} S(t)\\ S_v(t)\\ I(t)\\ I_v(t)\\ R(t)\\ R_v(t)\\ J(t)\\ J_v(t) \end{array}\right] ,\quad \varvec{G}(t,\varvec{z}(t))=\left[ \begin{array}{c} g_1(t)\\ g_2(t)\\ g_3(t)\\ g_4(t)\\ g_5(t)\\ g_6(t)\\ g_7(t)\\ g_8(t) \end{array}\right] = \left[ \begin{array}{c} \Lambda - \beta S(I+I_v)- (\lambda +\mu ) S+ \theta _1 R\\ -\beta ' S_v(I+ I_v)+ \theta _2R_v+ \lambda S- (\delta +\mu ) S_v\\ \beta S(I+ I_v)- (\gamma _1+\alpha _1+\mu ) I \\ \beta ' S_v(I+ I_v)- (\gamma _2+\alpha _2+\mu )I_v\\ \gamma _1 I-(\theta _1+\mu ) R+ \eta _1 J \\ \gamma _2 I_v- (\theta _2+\mu ) R_v+ \eta _2J_v+ \delta S_v\\ \alpha _1 I- (\eta _1+\mu _1)J\\ \alpha _2I_v- (\eta _2+\mu _2) J_v \end{array}\right] . \end{aligned}$$

To begin the QLM process, we assume $\varvec{z}_0(t)$ is available as an initial rough approximation for the solution $\varvec{z}(t)$ of the COVID-19 system Eq. (31). Through an iterative manner, the QLM procedure reads as follows

$$\begin{aligned} \frac{d}{dt}\varvec{z}_{s}(t)\approx \varvec{G}(t,\varvec{z}_{s-1}(t))+\varvec{G}_{\varvec{z}}(t,\varvec{z}_{s-1}(t))\,\left( \varvec{z}_{s}(t)-\varvec{z}_{s-1}(t)\right) ,\quad s=1,2,\ldots . \end{aligned}$$

Here, the notation $\varvec{G}_{\varvec{z}}$ stands for the Jacobian matrix of the COVID-19 system Eq. (31), which is of size 8 by 8. By performing some calculations we reach the linearized equivalent model form as

32 $$\begin{aligned} \frac{d}{dt}\varvec{z}_{s}(t)+\varvec{M}_{s-1}(t)\,\varvec{z}_{s}(t)=\varvec{r}_{s-1}(t),\qquad s=1,2,\ldots , \end{aligned}$$

where $\varvec{M}_{s-1}(t):=\varvec{J}(S_{s-1}(t), (S_v)_{s-1}(t), I_{s-1}(t), (I_v)_{s-1}(t))$ as the Jacobian matrix $\varvec{J}$ previously constructed in Eq. (7). Also we have

$$\begin{aligned} \varvec{z}_{s}(t)= \left[ \begin{array}{c} S_{s-1}(t)\\ (S_v)_{s-1}(t)\\ I_{s-1}(t)\\ (I_v)_{s-1}(t)\\ R_{s-1}(t)\\ (R_v)_{s-1}(t)\\ J_{s-1}(t)\\ (J_v)_{s-1}(t) \end{array}\right] ,\quad \varvec{r}_{s-1}(t)= \left[ \begin{array}{c} \Lambda +\beta \,S_{s-1}(t)\Big (I_{s-1}(t)+(I_v)_{s-1}(t)\Big )\\ \beta '\,(S_v)_{s-1}(t)\Big (I_{s-1}(t)+(I_v)_{s-1}(t)\Big )\\ -\beta \,S_{s-1}(t)\Big (I_{s-1}(t)+(I_v)_{s-1}(t)\Big )\\ -\beta '\,(S_v)_{s-1}(t)\Big (I_{s-1}(t)+(I_v)_{s-1}(t)\Big )\\ 0\\ 0\\ 0\\ 0 \end{array}\right] . \end{aligned}$$

Along with the system Eq. (32) the initial conditions

33 $$\begin{aligned} \varvec{z}_{s}(0)=\left[ \begin{array}{cccccccc} S_0&S_{v0}&I_0&I_{v0}&R_0&R_{v0}&J_0&J_{v0} \end{array}\right] ^T, \end{aligned}$$

are given due to Eq. (2). We now are able to solve the family of linearized initial-value problems Eqs. (32)-(33) numerically by our proposed matrix collocation method on an arbitrary (long) domain $[t_a,t_b]$. For this purpose and for clarity of exposition, we restrict our illustrations to a local subinterval $K_n$ for $n=0,1,\ldots ,N-1$.

In view of Eq. (29) by utilizing only ($\mathcal {J}+1$) SCPSK basis functions, we assume that the eight solutions of system Eq. (32) can be represented in terms of Eq. (29). Thus, we take these solutions at iteration $s\ge 1$ as

34 $$\begin{aligned} \left\{ \begin{array}{l} S^{n}_{\mathcal {J},s}(t)=\sum _{j=0}^{\mathcal {J}}\omega ^{n,s}_{j,1}\,\mathbb {U}_j(t)=\varvec{U}_{\mathcal {J}}(t)\,\varvec{W}^{n,s}_{\mathcal {J},1},\quad (S_v)^{n}_{\mathcal {J},s}(t)=\sum _{j=0}^{\mathcal {J}}\omega ^{n,s}_{j,2}\,\mathbb {U}_j(t)=\varvec{U}_{\mathcal {J}}(t)\,\varvec{W}^{n,s}_{\mathcal {J},2},\\ I^{n}_{\mathcal {J},s}(t)\,=\sum _{j=0}^{\mathcal {J}}\omega ^{n,s}_{j,3}\,\mathbb {U}_j(t)=\varvec{U}_{\mathcal {J}}(t)\,\varvec{W}^{n,s}_{\mathcal {J},3},\quad (I_v)^{n}_{\mathcal {J},s}(t)~=\sum _{j=0}^{\mathcal {J}}\omega ^{n,s}_{j,4}\,\mathbb {U}_j(t)=\varvec{U}_{\mathcal {J}}(t)\,\varvec{W}^{n,s}_{\mathcal {J},4},\\ R^{n}_{\mathcal {J},s}(t)=\sum _{j=0}^{\mathcal {J}}\omega ^{n,s}_{j,5}\,\mathbb {U}_j(t)=\varvec{U}_{\mathcal {J}}(t)\,\varvec{W}^{n,s}_{\mathcal {J},5},\quad (R_v)^{n}_{\mathcal {J},s}(t)=\sum _{j=0}^{\mathcal {J}}\omega ^{n,s}_{j,6}\,\mathbb {U}_j(t)=\varvec{U}_{\mathcal {J}}(t)\,\varvec{W}^{n,s}_{\mathcal {J},6},\\ J^{n}_{\mathcal {J},s}(t)\,=\sum _{j=0}^{\mathcal {J}}\omega ^{n,s}_{j,7}\,\mathbb {U}_j(t)=\varvec{U}_{J}(t)\,\varvec{W}^{n,s}_{\mathcal {J},7},\quad (J_v)^{n}_{\mathcal {J},s}(t)\,=\sum _{j=0}^{\mathcal {J}}\omega ^{n,s}_{j,8}\,\mathbb {U}_j(t)=\varvec{U}_{\mathcal {J}}(t)\,\varvec{W}^{n,s}_{\mathcal {J},8},\\ \end{array}\right. \end{aligned}$$

for $t\in K_n$. Moreover, by $$\varvec{W}^{n,s}_{\mathcal {J},i}= \left[ \begin{array}{cccc} \omega ^{n,s}_{0,i}&\omega ^{n,s}_{1,i}&\dots&\omega ^{n,s}_{\mathcal {J},i} \end{array}\right] ^T$$ we denote the vectors of unknowns for $1\le i\le 8$ at the iteration $s\ge 1$. Also, the vector of SCPSK basis, i.e., $\varvec{U}_\mathcal {J}(t)$ is defined in Eq. (29). We next provide a decomposition for $\varvec{U}_\mathcal {J}(t)$ given by

35 $$\begin{aligned} \varvec{U}_\mathcal {J}(t)=\varvec{Q}_\mathcal {J}(t)\,\varvec{F}_\mathcal {J}. \end{aligned}$$

Here, the vector $\varvec{Q}_\mathcal {J}(t)$ including the powers of $(t-t_n)$ introduced by

$$\begin{aligned} \varvec{Q}_\mathcal {J}(t)=\left[ 1\quad t-t_n\quad (t-t_n)^{2}\quad \ldots \quad (t-t_n)^{\mathcal {J}}\right] . \end{aligned}$$

The next object is the matrix $\varvec{F}_\mathcal {J}=(f_{i,j})_{i,j=0}^{\mathcal {J}}$ of size $(\mathcal {J}+1)\times (\mathcal {J}+1)$. The entries of the latter matrix are given in Eq. (15). One can also show that $\det (\varvec{F}_\mathcal {J})\ne 0$ and it is a triangular matrix. It follows that

$$\begin{aligned} f_{i,j}:= \left\{ \begin{array}{ll} o_{i,j}, &{} \textrm{if}~ i\le j,\\ 0, &{} \textrm{if}~ i> j. \end{array}\right. \end{aligned}$$

We then insert the obtained term $\varvec{U}_\mathcal {J}(t)$ in Eq. (35) into Eq. (34). The resulting expansions are

36 $$\begin{aligned} \left\{ \begin{array}{l} S^{n}_{\mathcal {J},s}(t)=\varvec{Q}_\mathcal {J}(t)\,\varvec{F}_\mathcal {J}\,\varvec{W}^{n,s}_{\mathcal {J},1},\quad (S_v)^{n}_{\mathcal {J},s}(t)=\varvec{Q}_\mathcal {J}(t)\,\varvec{F}_\mathcal {J}\,\varvec{W}^{n,s}_{\mathcal {J},2},\\ I^{n}_{\mathcal {J},s}(t)\,=\varvec{Q}_\mathcal {J}(t)\,\varvec{F}_J\,\varvec{W}^{n,s}_{\mathcal {J},3},\quad (I_v)^{n}_{\mathcal {J},s}(t)~=\varvec{Q}_\mathcal {J}(t)\,\varvec{F}_\mathcal {J}\,\varvec{W}^{n,s}_{\mathcal {J},4},\\ R^{n}_{\mathcal {J},s}(t) =\varvec{Q}_\mathcal {J}(t)\,\varvec{F}_\mathcal {J}\,\varvec{W}^{n,s}_{\mathcal {J},5},\quad (R_v)^{n}_{\mathcal {J},s}(t)=\varvec{Q}_\mathcal {J}(t)\,\varvec{F}_\mathcal {J}\,\varvec{W}^{n,s}_{\mathcal {J},6},\\ J^{n}_{\mathcal {J},s}(t)\, =\varvec{Q}_\mathcal {J}(t)\,\varvec{F}_\mathcal {J}\,\varvec{W}^{n,s}_{\mathcal {J},7},\quad (J_v)^{n}_{\mathcal {J},s}(t)\,=\varvec{Q}_\mathcal {J}(t)\,\varvec{F}_\mathcal {J}\,\varvec{W}^{n,s}_{\mathcal {J},8}, \end{array}\right. t\in K_n. \end{aligned}$$

We then proceed by nothing that the derivative of the vector $\varvec{Q}_\mathcal {J}(t)$ can be stated in terms of itself. A vivid calculation reveals that

37 $$\begin{aligned} \dot{\varvec{Q}}_{\mathcal {J}}(t)=\varvec{Q}_{\mathcal {J}}(t)\,\varvec{D}_\mathcal {J},\quad \varvec{D}_\mathcal {J}=\left[ \begin{array}{lllll} 0 &{} 1 &{} 0 &{}\ldots &{} 0\\ 0 &{} 0 &{} 2 &{}\ldots &{} 0\\ \vdots &{} \vdots &{} \ddots &{}\vdots &{} \vdots \\ 0 &{} 0 &{} 0 &{}\ddots &{} \mathcal {J}\\ 0 &{} 0 &{} 0 &{} \ldots &{} 0 \end{array}\right] _{(\mathcal {J}+1)\times (\mathcal {J}+1)}. \end{aligned}$$

From this relation, we are able to derive a matrix forms of the derivatives of the unknown solutions in Eq. (36).

38 $$\begin{aligned} \left\{ \begin{array}{l} \dot{S}^{n}_{\mathcal {J},s}(t)=\varvec{Q}_\mathcal {J}(t)\,\varvec{D}_\mathcal {J}\,\varvec{F}_\mathcal {J}\,\varvec{W}^{n,s}_{\mathcal {J},1},\quad (\dot{S}_v)^{n}_{\mathcal {J},s}(t)=\varvec{Q}_\mathcal {J}(t)\,\varvec{D}_\mathcal {J}\,\varvec{F}_\mathcal {J}\,\varvec{W}^{n,s}_{\mathcal {J},2},\\ \dot{I}^{n}_{\mathcal {J},s}(t)\,=\varvec{Q}_\mathcal {J}(t)\,\varvec{D}_\mathcal {J}\,\varvec{F}_\mathcal {J}\,\varvec{W}^{n,s}_{\mathcal {J},3},\quad (\dot{I}_v)^{n}_{\mathcal {J},s}(t)~=\varvec{Q}_\mathcal {J}(t)\,\varvec{D}_\mathcal {J}\,\varvec{F}_\mathcal {J}\,\varvec{W}^{n,s}_{\mathcal {J},4},\\ \dot{R}^{n}_{\mathcal {J},s}(t)=\varvec{Q}_\mathcal {J}(t)\,\varvec{D}_\mathcal {J}\,\varvec{F}_\mathcal {J}\,\varvec{W}^{n,s}_{\mathcal {J},5},\quad (\dot{R}_v)^{n}_{\mathcal {J},s}(t)=\varvec{Q}_\mathcal {J}(t)\,\varvec{D}_\mathcal {J}\,\varvec{F}_\mathcal {J}\,\varvec{W}^{n,s}_{\mathcal {J},6},\\ \dot{J}^{n}_{\mathcal {J},s}(t)\,=\varvec{Q}_\mathcal {J}(t)\,\varvec{D}_\mathcal {J}\,\varvec{F}_\mathcal {J}\,\varvec{W}^{n,s}_{\mathcal {J},7},\quad (\dot{J}_v)^{n}_{\mathcal {J},s}(t)\,=\varvec{Q}_\mathcal {J}(t)\,\varvec{D}_\mathcal {J}\,\varvec{F}_\mathcal {J}\,\varvec{W}^{n,s}_{\mathcal {J},8}, \end{array}\right. t\in K_n. \end{aligned}$$

The exact solutions of the linearized system Eq. (32) can be written in a vectorized form as

39 $$\begin{aligned} \varvec{z}_s(t)\approx \varvec{z}^n_{\mathcal {J},s}(t):= \left[ \begin{array}{l} S^{n}_{\mathcal {J},s}(t)\\ (S_v)^{n}_{\mathcal {J},s}(t)\\ I^{n}_{\mathcal {J},s}(t)\\ (I_v)^{n}_{\mathcal {J},s}(t)\\ R^{n}_{\mathcal {J},s}(t)\\ (R_v)^{n}_{\mathcal {J},s}(t)\\ J^{n}_{\mathcal {J},s}(t)\\ (J_v)^{n}_{\mathcal {J},s}(t) \end{array}\right] ,\quad \dot{\varvec{z}}_s(t)\approx \frac{d}{dt}\varvec{z}^n_{\mathcal {J},s}(t):= \left[ \begin{array}{l} \dot{S}^{n}_{\mathcal {J},s}(t)\\ (\dot{S}_v)^{n}_{\mathcal {J},s}(t)\\ \dot{I}^{n}_{\mathcal {J},s}(t)\\ (\dot{I}_v)^{n}_{\mathcal {J},s}(t)\\ \dot{R}^{n}_{\mathcal {J},s}(t)\\ (\dot{R}_v)^{n}_{\mathcal {J},s}(t)\\ \dot{J}^{n}_{\mathcal {J},s}(t)\\ (\dot{J}_v)^{n}_{\mathcal {J},s}(t) \end{array}\right] . \end{aligned}$$

We next introduce the following block diagonal matrices of dimensions $8(\mathcal {J}+1)\times 8(\mathcal {J}+1)$ as

$$\begin{aligned} \widehat{\varvec{Q}}(t){} & {} =\mathrm {{\textbf {Diag}}} \left( \begin{array}{cccccccc} \varvec{Q}_\mathcal {J}(t)&\varvec{Q}_\mathcal {J}(t)&\varvec{Q}_\mathcal {J}(t)&\varvec{Q}_\mathcal {J}(t)&\varvec{Q}_\mathcal {J}(t)&\varvec{Q}_\mathcal {J}(t)&\varvec{Q}_\mathcal {J}(t)&\varvec{Q}_\mathcal {J}(t) \end{array}\right) ,\\ \widehat{\varvec{D}}{} & {} =\mathrm {{\textbf {Diag}}} \left( \begin{array}{cccccccc} \varvec{D}_\mathcal {J}&\varvec{D}_\mathcal {J}&\varvec{D}_\mathcal {J}&\varvec{D}_\mathcal {J}&\varvec{D}_\mathcal {J}&\varvec{D}_\mathcal {J}&\varvec{D}_\mathcal {J}&\varvec{D}_\mathcal {J} \end{array}\right) ,\\ \widehat{\varvec{F}}{} & {} =\mathrm {{\textbf {Diag}}} \left( \begin{array}{cccccccc} \varvec{F}_\mathcal {J}&\varvec{F}_\mathcal {J}&\varvec{F}_\mathcal {J}&\varvec{F}_\mathcal {J}&\varvec{F}_\mathcal {J}&\varvec{F}_\mathcal {J}&\varvec{F}_\mathcal {J}&\varvec{F}_\mathcal {J} \end{array}\right) . \end{aligned}$$

By the aid of the former definitions, the matrix formats of $\varvec{z}^n_{\mathcal {J},s}(t)$ and $\dot{\varvec{z}}^n_{\mathcal {J},s}(t)$ will rewrite concisely as

40 $$\begin{aligned} \varvec{z}^n_{\mathcal {J},s}(t)=\widehat{\varvec{Q}}(t)\,\widehat{\varvec{F}}\,\varvec{W}^n,\quad \dot{\varvec{z}}^n_{\mathcal {J},s}(t)=\widehat{\varvec{Q}}(t)\,\widehat{\varvec{F}}\,\widehat{\varvec{D}}\,\varvec{W}^n. \end{aligned}$$

Here, $\varvec{W}^n$ is the successive vector of eight previously defined vector of unknowns

$$\begin{aligned} \varvec{W}^n=\left[ \begin{array}{cccc} \varvec{W}^{n,s}_{\mathcal {J},1}&\varvec{W}^{n,s}_{\mathcal {J},2}&\ldots&\varvec{W}^{n,s}_{\mathcal {J},8} \end{array}\right] ^T. \end{aligned}$$

We now can collocate the linearized Eq. (32) at the zeros of SCPSK given in Eq. (17) on the subdomain $K_n$. We get

41 $$\begin{aligned} \frac{d}{dt}\varvec{z}_{s}(t_{\nu ,n})+\varvec{M}_{s-1}(t_{\nu ,n})\,\varvec{z}_{s}(t_{\nu ,n})=\varvec{r}_{s-1}(t_{\nu ,n}),\qquad \nu =1,2,\ldots ,\mathcal {J}, \end{aligned}$$

for $s=1,2,\ldots$. Denote the coefficient matrix by $\widehat{\varvec{M}}^n_{s-1}$ and the right-hand-side vector as $\widehat{\varvec{R}}^n_{s-1}$. These are defined by

$$\begin{aligned} \widehat{\varvec{M}}^n_{s-1}= \left[ \begin{array}{cccc} \varvec{M}_{s-1}(t_{0,n})&{}\textbf{0}&{}\ldots &{}\textbf{0}\\ \textbf{0}&{}\varvec{M}_{s-1}(t_{1,n})&{}\ldots &{}\textbf{0}\\ \vdots &{}\vdots &{}\ddots &{}\vdots \\ \textbf{0}&{}\textbf{0}&{}\ldots &{}\varvec{M}_{s-1}(t_{\mathcal {J},n}) \end{array}\right] ,\quad \widehat{\varvec{R}}^n_{s-1}= \left[ \begin{array}{c} \varvec{r}_{s-1}(t_{0,n})\\ \varvec{r}_{s-1}(t_{1,n})\\ \vdots \\ \varvec{r}_{s-1}(t_{\mathcal {J},n}) \end{array}\right] . \end{aligned}$$

Let us define further the vectors of unknowns as

$$\begin{aligned} \dot{\varvec{Z}}^n_s= \left[ \begin{array}{c} \dot{\varvec{z}}_{s}(t_{0,n})\\ \dot{\varvec{z}}_{s}(t_{1,n})\\ \vdots \\ \dot{\varvec{z}}_{s}(t_{\mathcal {J},n}) \end{array}\right] ,\quad \varvec{Z}^n_s= \left[ \begin{array}{c} \dot{\varvec{z}}_{s}(t_{0,n})\\ \dot{\varvec{z}}_{s}(t_{1,n})\\ \vdots \\ \dot{\varvec{z}}_{s}(t_{\mathcal {J},n}) \end{array}\right] . \end{aligned}$$

Consequently, the system of Eq. (41) can be stated briefly as

42 $$\begin{aligned} \dot{\varvec{Z}}^{n}_{s}+\widehat{\varvec{M}}^n_{s-1}\,\varvec{Z}^n_s=\widehat{\varvec{R}}^n_{s-1},\quad n=0,1,\ldots ,N-1, \end{aligned}$$

and with $s=1,2,\ldots$. Before we talk about the fundamental matrix equation, we need to state two vectors $\varvec{Z}^n_s$ and $\dot{\varvec{Z}}^{n}_{s}$ in Eq. (42) in the matrix representation forms. The proof is easy by just considering the definitions of the involved matrices and vectors in Eq. (40).

#### Lemma 4.1

If two vectors $\varvec{z}^n_{\mathcal {J},s}(t)$ and $\dot{\varvec{z}}^n_{\mathcal {J},s}(t)$ in Eq. (40) computed at the collocation points Eq. (30), we arrive at the next matrix forms

43 $$\begin{aligned} \varvec{Z}^n_s=\bar{\widehat{\varvec{Q}}}\,\widehat{\varvec{F}}\,\varvec{W}^n,\qquad \dot{\varvec{Z}}^n_s=\bar{\widehat{\varvec{Q}}}\,\widehat{\varvec{F}}\,\widehat{\varvec{D}}\,\varvec{W}^n, \end{aligned}$$

where the matrix $\bar{\widehat{\varvec{Q}}}$ is given by

$$\begin{aligned} \bar{\widehat{\varvec{Q}}}=[\widehat{\varvec{Q}}(t_{0,n})\quad \widehat{\varvec{Q}}(t_{1,n})\quad \ldots \quad \widehat{\varvec{Q}}(t_{\mathcal {J},n}) ]^T. \end{aligned}$$

Moreover, two matrices $\widehat{\varvec{Q}}, \widehat{\varvec{F}}$ are defined in Eq. (40). Similarly, the vector $\varvec{W}^n$ is given in Eq. (40).

By turning to relation Eq. (40) we substitute the derived matrix formats into it. Precisely speaking, after replacing $\varvec{Z}^n_s$ and $\dot{\varvec{Z}}^n_s$ we gain the so-called fundamental matrix equation (FME) of the form

44 $$\begin{aligned} \varvec{B}_n\,\varvec{W}^n=\widehat{\varvec{R}}^n_{s-1}, \quad \textrm{or}\quad \left[ \varvec{B}_n;\widehat{\varvec{R}}^n_{s-1}\right] ,\quad s\ge 1,~0\le n\le N-1, \end{aligned}$$

where

$$\begin{aligned} \varvec{B}_n:=\bar{\widehat{\varvec{Q}}}\,\widehat{\varvec{F}}+\widehat{\varvec{M}}^n_{s-1}\,\bar{\widehat{\varvec{Q}}}\,\widehat{\varvec{F}}\,\widehat{\varvec{D}}. \end{aligned}$$

To complete the process of QLM-SCPSK approach, it is necessary to implement the initial conditions in Eq. (2) and add them into Eq. (44). So, the next task is to constitute the matrix representation of Eq. (2). Let us approach $t\rightarrow 0$ in the first relation of Eq. (40). It gives us

$$\begin{aligned} \varvec{B}_{0,n}\,\varvec{W}^n=\widehat{\varvec{R}}^n_{s-1,0},\qquad \varvec{B}_{0,n}:=\widehat{\varvec{Q}}(0)\,\widehat{\varvec{F}},\quad \widehat{\varvec{R}}^n_{s-1,0}=\left[ \begin{array}{cccccccc} S_0&S_{v0}&I_0&I_{v0}&R_0&R_{v0}&J_0&J_{v0} \end{array}\right] ^T. \end{aligned}$$

We then replace eight rows of the augmented matrix $[\varvec{B}_n;\widehat{\varvec{R}}^n_{s-1}]$ by the already obtained row matrix $[\varvec{B}_{0,n};\widehat{\varvec{R}}^n_{s-1,0}]$. Denote the modified FME by

45 $$\begin{aligned} \check{\varvec{B}_{n}}\,\varvec{W}^n=\check{\textbf{R}}^n_{s-1},\quad \textrm{or} \quad \left[ \check{\varvec{B}_{n}};\check{\textbf{R}}^n_{s-1}\right] . \end{aligned}$$

This implies that the solution of the model Eq. (1) is obtainable on each subdomain $K_n$ by iterating $n=0,1,\ldots ,N-1$. On $K_0$ as the first subdomain, the given initial conditions in Eq. (2) will be used to find the corresponding approximations for the system Eq. (1). Hence, this approximate solutions on $K_0$ evaluated at the starting point of $K_1$ will be utilized for the initial conditions on $K_1$. By repeating this process we acquire all approximations on all $K_n$ for $0\le n\le N-1$.

### REFs and QLM-SCPSK technique

Generally, finding the true solutions of the COVID-19 system Eq. (1) is not possible practically. In this case, the residual error functions (REFs) help us to measure the quality of approximations obtained by the QLM-SCPSK technique. Once we calculate the eight approximations by the illustrated method, we substitute them into the model system Eq. (1). In fact, the REFs are defined as the difference between the left-hand side and the right-hand side of the considered equation. On the subdomain $K_n$ we set the REFs as

46 $$\begin{aligned}{} & {} \mathbb {R}_{1,\mathcal {J}}^{n}(t):=\left| \dot{S}^{n}_{\mathcal {J},s}(t)-\Lambda +\beta S^{n}_{\mathcal {J},s}(t)L^n_{\mathcal {J},s}(t)+(\lambda +\mu ) S^{n}_{\mathcal {J},s}(t)- \theta _1 R^{n}_{\mathcal {J},s}(t)\right| \cong 0, \nonumber \\{} & {} \mathbb {R}_{2,\mathcal {J}}^{n}(t):=\left| (\dot{S_v})^{n}_{\mathcal {J},s}(t)+\beta ' (S_v)^{n}_{\mathcal {J},s}(t)L^n_{\mathcal {J},s}(t)- \theta _2(R_v)^{n}_{\mathcal {J},s}(t)- \lambda S^{n}_{\mathcal {J},s}(t)+ (\delta +\mu ) (S_v)^{n}_{\mathcal {J},s}(t)\right| \cong 0, \nonumber \\{} & {} \mathbb {R}_{3,\mathcal {J}}^{n}(t):=\left| \dot{I}^{n}_{\mathcal {J},s}(t)-\beta S^{n}_{\mathcal {J},s}(t)L^n_{\mathcal {J},s}(t)+ (\gamma _1+\alpha _1+\mu ) I^{n}_{\mathcal {J},s}(t)\right| \cong 0, \nonumber \\{} & {} \mathbb {R}_{4,\mathcal {J}}^{n}(t):=\left| (\dot{I_v})^{n}_{\mathcal {J},s}(t)- \beta ' (S_v)^{n}_{\mathcal {J},s}(t)L^n_{\mathcal {J},s}(t)+ (\gamma _2+\alpha _2+\mu )(I_v)^{n}_{\mathcal {J},s}(t)\right| \cong 0, \nonumber \\{} & {} \mathbb {R}_{5,\mathcal {J}}^{n}(t):=\left| \dot{R}^{n}_{\mathcal {J},s}(t) - \gamma _1 I^{n}_{\mathcal {J},s}(t)+(\theta _1+\mu ) R^{n}_{\mathcal {J},s}(t)- \eta _1 J^{n}_{\mathcal {J},s}(t)\right| \cong 0, \nonumber \\{} & {} \mathbb {R}_{6,\mathcal {J}}^{n}(t):=\left| (\dot{R_v})^{n}_{\mathcal {J},s}(t)- \gamma _2 (I_v)^{n}_{\mathcal {J},s}(t)+ (\theta _2+\mu ) (R_v)^{n}_{\mathcal {J},s}(t)- \eta _2(J_v)^{n}_{\mathcal {J},s}(t)- \delta (S_v)^{n}_{\mathcal {J},s}(t)\right| \cong 0, \nonumber \\{} & {} \mathbb {R}_{7,\mathcal {J}}^{n}(t):=\left| \dot{J}^{n}_{\mathcal {J},s}(t) -\alpha _1 I^{n}_{\mathcal {J},s}(t)+ (\eta _1+\mu _1)J^{n}_{\mathcal {J},s}(t)\right| \cong 0, \nonumber \\{} & {} \mathbb {R}_{8,\mathcal {J}}^{n}(t):=\left| (\dot{J_v})^{n}_{\mathcal {J},s}(t)- \alpha _2(I_v)^{n}_{\mathcal {J},s}(t) +(\eta _2+\mu _2) (J_v)^{n}_{\mathcal {J},s}(t)\right| \cong 0, \end{aligned}$$

for a fixed iteration number *s* and we have defined $L^n_{\mathcal {J},s}:=I^{n}_{\mathcal {J},s}(t)+ (I_v)^{n}_{\mathcal {J},s}(t)$ for brevity.

Analogously, at the fixed iteration *s*, the numerical order of convergence associated with the obtained REFs can be defined in the infinity norm. These are given by

$$\begin{aligned} L^{\infty }_{\ell }\equiv L^{\infty }_{\ell }(\mathcal {J}):=\max _{0\le n\le N-1}\left( \max _{t\in K_n}\,\left| \mathbb {R}_{\ell ,\mathcal {J}}^{n}(t)\right| \right) ,\quad \ell =1,2,\ldots ,8. \end{aligned}$$

Therefore, the convergence order (Co) for each solution is defined by

47 $$\begin{aligned} \textrm{Co}_{\mathcal {J}}^{\ell }:=\log _2\left( \frac{L^{\infty }_{\ell }(\mathcal {J})}{L^{\infty }_{\ell }(2\mathcal {J})}\right) ,\quad \ell =1,2,\ldots ,8. \end{aligned}$$

## Experimental calculations

We now exploit the proposed QLM-SCPSK collocation technique to solve the COVID-19 system Eq. (1) numerically. We use Matlab version 2021a installed on a personnel laptop to run our algorithm. Different values of the model parameters will be utilized in the experimental results. Setting $s=5$ is sufficient in the iterated QLM to reach the desired accuracy. For each run, to begin computations we use $\varvec{z}_0(t)\equiv 0$ as the first rough approximation for the linearized system Eq. (32).

To solve the underlying COVID-19 model, we need to determine the involved parameters in the model. Towards this end, the estimations of these parameters are done in accordance with the statistics given by the Kerman University of Medical Sciences (KUMS) during the period between 22 December 2021 and 5 May 2022. The estimated parameters are listed in Table 1.

**Table 1:** Parameters: Descriptions and their values

Parameter	Description	Value
$\alpha _1:$	The rate of unvaccinated infected people and are isolated	0.2
$\alpha _2:$	The rate of vaccinated infected people and are isolated	$\frac{\alpha _1}{30}$
$\beta :$	Transmission rate for unvaccinated individuals	$3.6\times 10^{-7}$
$\beta ':$	Transmission rate for vaccinated individuals	$\frac{\beta }{30}$
$\eta _1:$	Recovery rate in class *J*	$\frac{1}{7}$
$\eta _2:$	Recovery rate in class $J_v$	$\frac{1}{7}$
$\delta :$	A fraction of vaccinated susceptible goes to $R_v$ class	0.997
$\gamma _1:$	The recovery rate of infected people who did not receive the vaccine	$\frac{1}{7}$
$\gamma _2:$	The recovery rate of infected people who received the vaccine	$\frac{1}{7}$
$\mu :$	Natural mortality rate	0.008
$\mu _1:$	Death rate due to disease in isolated people without vaccine	0.02
$\mu _2:$	Death rate due to disease in isolated people with vaccine	0.01
$\theta _1:$	The rate of reinfection in class *R*	$\frac{1}{60}$
$\theta _2:$	The rate of reinfection in class $R_v$	$\frac{1}{60}$
$\Lambda :$	Population growth rate	0.8

The initial conditions are taken as

48 $$\begin{aligned} \left\{ \begin{array}{ll} S_0=1184950,\quad S_{v0}=1815050,\quad I_0=760,\quad I_{v0}=25,\\ R_0=2720,\quad R_{v0}=25,\quad J_0=152,\quad J_{v0}=5. \end{array}\right. \end{aligned}$$

**Fig. 2:**
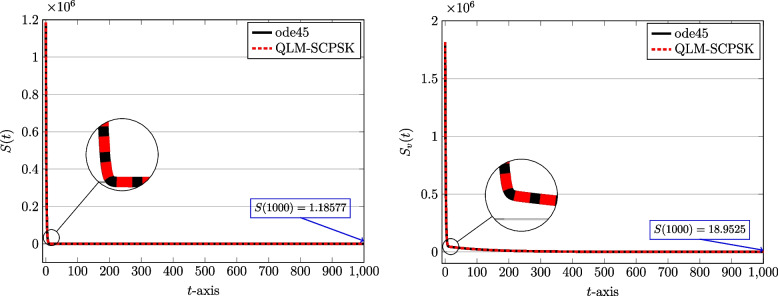
The behavior of susceptible population: Unvaccinated *S*(*t*) (left) and vaccinated $S_v(t)$ (right) obtained via QLM-SCPSK methodology using $\mathcal {J}=10$ and $N=100$ on [0, 1000]

**Fig. 3:**
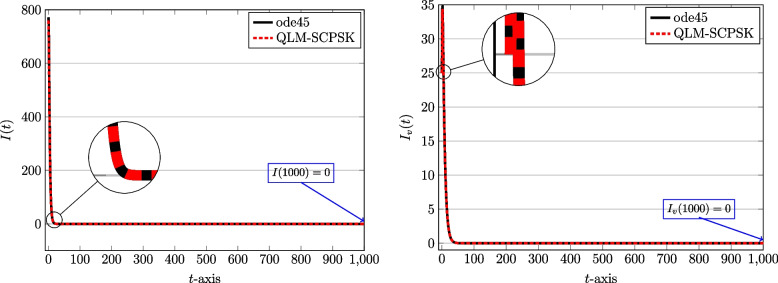
The behavior of infected population: Unvaccinated *I*(*t*) (left) and vaccinated $I_v(t)$ (right) obtained via QLM-SCPSK methodology using $\mathcal {J}=10$ and $N=100$ on [0, 1000]

**Fig. 4:**
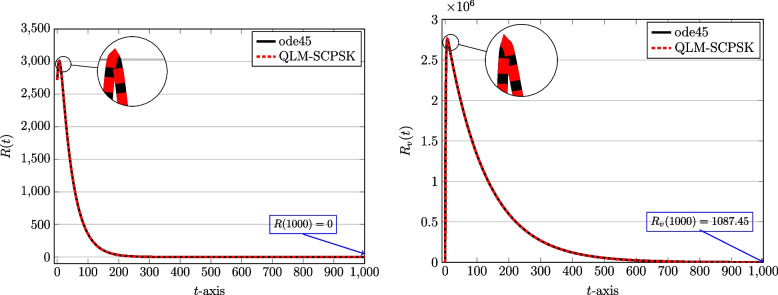
The behavior of recovered population: Unvaccinated *R*(*t*) (left) and vaccinated $R_v(t)$ (right) obtained via QLM-SCPSK methodology using $J=10$ and $N=100$ on [0, 1000]

**Fig. 5:**
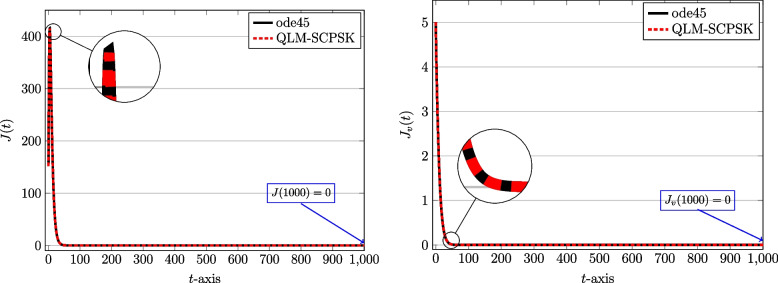
The behavior of isolated population: Unvaccinated *J*(*t*) (left) and vaccinated $J_v(t)$ (right) obtained via QLM-SCPSK methodology using $\mathcal {J}=10$ and $N=100$ on [0, 1000]

To start computations, we first set $J=10$ and the computational domain is taken as $[t_a,t_b]=[0,1000]$ in terms of days spent during the pandemic. The number of subintervals used is $N=100$. Through Figs. 2, 3, 4 and 5 we show the behaviors of the approximate solutions obtained via QLM-SCPSK procedure for susceptible, infected, recovered, and isolated populations in both unvaccinated and vaccinated parts. These curves are presented by dashed red lines. Besides, the outcomes of the well-known Matlab function ode45 are also depicted to validate our approximations. These plots are shown by solid black lines. As one sees the plots obtained by both methods are matched very well. More accuracy is attainable by just increasing the number of bases, namely *J*, or using a larger number of subintervals *N* in the computations. Note that for each solution the value of the corresponding population at the endpoint $t=1000$ is given for completeness.

As you see, the population of susceptible individuals will decrease in both vaccinated and unvaccinated groups. But with consideration, so a large fraction of the population receives the vaccine, a large number of people directly enter the group of recovered people $(R_v)$. Hence, the number of infected and isolated individuals is not high compared to the population and a significant decrease is observed in both vaccinated and unvaccinated groups. According to the prediction of these graphs, with the passage of time, the population goes through an epidemic, which is consistent with the reality that happened in the province of Kerman.

**Fig. 6:**
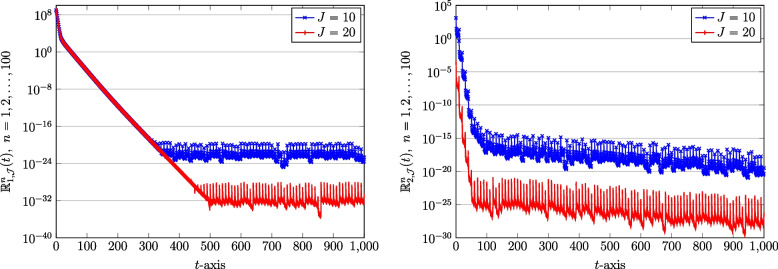
The REFs related to susceptible population: Unvaccinated $\mathbb {R}_{1,\mathcal {J}}^{n}(t)$ (left) and vaccinated $\mathbb {R}_{2,\mathcal {J}}^{n}(t)$ (right) obtained via QLM-SCPSK methodology using $\mathcal {J}=10,20$ and fixed $N=100$ on [0, 1000]

**Fig. 7:**
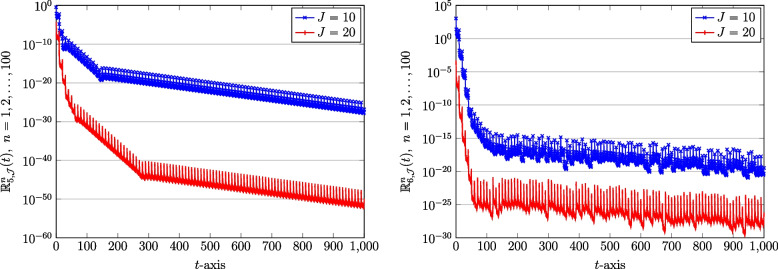
The REFs related to recovered population: Unvaccinated $\mathbb {R}_{5,\mathcal {J}}^{n}(t)$ (left) and vaccinated $\mathbb {R}_{6,\mathcal {J}}^{n}(t)$ (right) obtained via QLM-SCPSK methodology using $\mathcal {J}=10,20$ and fixed $N=100$ on [0, 1000]

We next show the effect of utilizing a diverse number of bases, $J=10,20$, on the computed solutions. In this respect, we compute the REFs formulae in Eq. (46) related to the approximate solutions of the COVID-19 model Eq. (1). To save space, we only visualize the REFs corresponding to the susceptible and recovered populations. These results for both vaccinated and unvaccinated counterparts are displayed in Figs. 6 and 7. Not that we used the fixed number of subintervals as $N=100$. It can clearly seen that the desirable form of accuracy is attainable if one increases the number of bases accordingly.

**Fig. 8:**
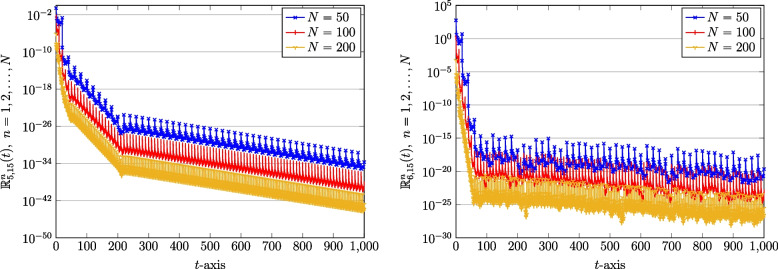
The REFs related to recovered population: Unvaccinated $\mathbb {R}_{5,\mathcal {J}}^{n}(t)$ (left) and vaccinated $\mathbb {R}_{6,\mathcal {J}}^{n}(t)$ (right) obtained via QLM-SCPSK methodology using a fixed $J=15$ and various $N=50,100,200$ on [0, 1000]

As we mentioned, we can also get the smaller magnitude of REFs by increasing the number of subintervals *N* in the proposed domain decomposition spectral QLM-SCPSK approach. Towards this end, let use take $N=50,100$ and $N=200$. For a fixed $J=15$, we show the results of REFs associated with the recovered populations (vaccinated and unvaccinated) in Fig. 8. From the plots shown in Fig. 8, it can be obviously concluded that high-order accuracy is attained for smaller lengths of intervals.

Finally, we compute $L^{\infty }_\ell$ error norms and the related numerical order of accuracy as shown by $\textrm{Co}_{\mathcal {J}}^\ell$ for $\ell =5,6,7,8$, namely for recovered and isolated populations. The results by using diverse values of $\mathcal {J}=2^k,$ for $k=1,2,\ldots ,5$ are listed in Table 2. For this purpose, we use $N=10$ and the computational interval is set as [0, 100]. By looking at the results tabulated in this table one can infer that the proposed spectral QLM-SCPSK approach has an exponential order of accuracy.

**Table 2:** Maximum value of REFs and the associated $\textrm{Co}_{\mathcal {J}}^{\ell }$ for $\ell =5,6,7,8$ obtained by using the QLM-SCPSK approach with $N=10$ and various $\mathcal {J}$ on [0, 100]

$\mathcal {J}$	$L^{\infty }_5$	$\textrm{Co}_{\mathcal {J}}^5$	$L^{\infty }_6$	$\textrm{Co}_{\mathcal {J}}^6$	$L^{\infty }_7$	$\textrm{Co}_{\mathcal {J}}^7$	$L^{\infty }_8$	$\textrm{Co}_{\mathcal {J}}^8$
2	$1.7318_{+01}$	−	$1.1180_{+06}$	−	$1.4769_{+01}$	−	$1.1180_{+06}$	−
4	$1.6228_{+01}$	0.0938	$3.9021_{+05}$	1.5186	$1.5071_{-03}$	13.2585	$3.9021_{+05}$	1.5186
8	$1.7063_{-01}$	6.5715	$2.3252_{+03}$	7.3907	$4.3107_{-12}$	28.3812	$2.3252_{+03}$	7.3907
16	$1.6259_{-03}$	6.7135	$2.6979_{-01}$	13.073	$5.9301_{-19}$	22.7934	$2.6979_{-01}$	13.073
32	$2.4221_{-11}$	26.000	$4.3585_{-11}$	32.527	$4.8004_{-34}$	50.1338	$4.3585_{-11}$	32.527

### Parameter study

**Fig. 9:**
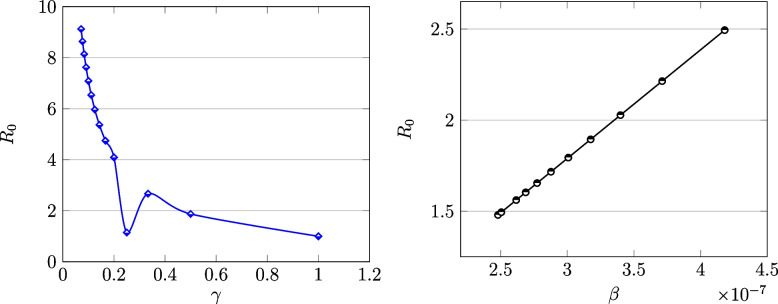
Plot of $R_0$ versus $\gamma$ (left) and the visualization of $R_0$ versus $\beta$ (right) in the COVID model

In this part, the relationship between $\beta$,  $\gamma$ and $R_0$ is examined. As you can see in the next Fig. 9 (left picture), as the $\gamma$ value increases, $R_0$ decreases. The lowest value of $R_0$ is at $\gamma =0.3$. Therefore, if the recovery rate reaches 0.3, the epidemic will disappear. The relationship between $\beta$ and $R_0$ is shown in the same figure but on the right panel. As you see, there is a direct relationship between them.

## Conclusions

A novel mathematical model for studying the COVID-19 pandemic disease has been suggested by dividing the population into vaccine and nonvaccine groups. From a dynamical point of view, the boundedness of the system was proved and the basic reproduction number was obtained to prove the stability of the equilibrium points. Moreover, the existence of bifurcation for the COVID-19 system was discussed. To get the solutions of this system, an efficient spectral method based on the shifted Chebyshev polynomials of the second kind (SCPSK) combined with the quasilinearization methodology (QLM) was used. The methodology of domain-splitting further was implemented to keep the accuracy of the proposed QLM-SCPSK approach at a desired level on a long time interval. The convergence analysis of the SCPSK basis functions in the $L_{\infty }$ norm was performed and an upper bound estimation for the error was done. The involved parameters were estimated by the real data provided by the Kerman University of Medical Sciences during the period between 22 December 2021 and 5 May 2022. Numerous computational experiments based on the proposed QLM-SCPSK technique were conducted to predict the behavior of disease over a long time interval. The presented QLM-SCPSK technique with high accuracy and efficacy can be generalized to solve similar epidemiological models with integer-order and fractional-order derivatives and even with more than eight equations.

## Data Availability

Data sharing not applicable to this article as no datasets were generated or analyzed during the current study.
